# Evaluation of the Efficacy and Effects of Common Hepatic Artery Reconstruction in Pancreas Transplantation: A Randomized Controlled Trial

**DOI:** 10.3390/jcm11082258

**Published:** 2022-04-18

**Authors:** Naohiro Aida, Taihei Ito, Kei Kurihara, Izumi Hiratsuka, Megumi Shibata, Atsushi Suzuki, Takashi Kenmochi

**Affiliations:** 1 Department of Transplantation and Regenerative Medicine, School of Medicine, Fujita Health University, 1-98, Dengakugakubo, Kutsukake-cho, Toyoake 470-1192, Japan; i-taihei@fujita-hu.ac.jp (T.I.); kurihara@fujita-hu.ac.jp (K.K.); kenmochi@fujita-hu.ac.jp (T.K.); 2 Department of Endocrinology, Diabetes and Metabolism, School of Medicine, Fujita Health University, 1-98, Dengakugakubo, Kutsukake-cho, Toyoake 470-1192, Japan; idumi0630@yahoo.co.jp (I.H.); megumi03@fujita-hu.ac.jp (M.S.); aslapin@fujita-hu.ac.jp (A.S.)

**Keywords:** pancreas transplantation, postoperative complications, reconstructive surgical procedures, graft survival, perfusion imaging

## Abstract

Maintenance of postoperative graft flow is important in pancreas transplantation. In Japan, reconstruction of the common hepatic artery is performed primarily to increase perfusion in the pancreatic head. We investigated the effects of common hepatic artery reconstruction on patient and graft survival and endocrine functions. Twenty-nine cases of pancreas transplantation were registered in the clinical trial. Of the 29 cases, four were excluded because of the risk of ischemia without reconstruction or complicated reconstruction due to a narrow artery. A total of 25 cases were randomized into two groups: 13 in the non-reconstructed group and 12 in the reconstructed group. The 1-year patient survival and graft survival rates of the non-reconstructed and reconstructed groups were 92.3% and 83.3%, and 91.7% and 82.5%, respectively. The incidence of complications in the two groups was comparable, with 38.5% (5/13 cases) in the non-reconstructed group and 33.3% (4/12 cases) in the reconstructed group. The results of the glucagon stimulation test and oral glucose tolerance test at 1 month and 1 year post-transplantation were comparable. Common hepatic artery reconstruction is not essential unless there is risk of ischemia. This study was registered at the University Hospital Medical Information Network Clinical Trials Registry under UMIN000027213.

## 1. Introduction

Pancreas transplantation is a curative treatment for type 1 diabetes mellitus. The number of pancreas transplantations in Japan has been increasing annually, and it is now a commonly accepted therapy [1]. It is said to have the highest complication rate among organ transplants [2]. Graft venous thrombosis and graft duodenal perforation are frequent complications and are the major causes of graft loss. Various risk factors have been reported for both complications, and their etiologies are thought to be multifactorial [3,4,5]. The hemodynamic status of the graft is an important risk factor as ischemia-reperfusion injury is inevitable during the process of procurement, storage, and transplantation. This results in hemodynamic fluctuations after transplantation. The pancreas is known to be extremely susceptible to ischemia-reperfusion injury [6], which affects its endocrine and exocrine functions as well as microcirculation. Benz et al. reported that reperfusion immediately after transplantation [7] and post-transplantation management were essential, in addition to excellent surgical technique. Hypotension and hypovolemia need to be avoided without the use of high doses of catecholamines for the stabilization of circulation in the early stages. In addition to this technical aspect, revascularization of the common hepatic artery (CHA) has been performed in Japan to increase perfusion in the head of the pancreas. This traditional procedure has a unique background related to the history of transplantation in Japan. First, since the history of brain-dead pancreas transplantation in Japan is short and the number of donors is small, considerable efforts have been made to achieve better results. Increased blood flow can reduce the frequency of ischemia-reperfusion injury and other complications, especially duodenal perforation and bleeding [8]. Second, in Japan, the liver and pancreas are usually procured simultaneously from the same donor. The arteries of the liver graft are dissected at the CHA and do not require the celiac trunk. Therefore, in most cases, the celiac trunk is attached to the pancreatic graft as a Carrel patch and does not require arterial reconstruction with a Y graft, making it possible to reconstruct the blood circulation from the celiac trunk to the gastroduodenal artery (GDA). This procedure is widely practiced in Japan. As the number of transplants increased, the outcomes of pancreas transplantation in Japan have also improved [1,9]. To further improve outcomes, the surgical procedures need to be refined.

In general, the pancreatic head region is perfused by the GDA branching from the CHA and the inferior pancreaticoduodenal artery (IPDA), which is a branch of the superior mesenteric artery (SMA), and these arteries often communicate with each other [10]. Theoretically, sufficient blood flow should reach the head of the pancreas from the IPDA without CHA reconstruction. However, the impact of CHA reconstruction has not been investigated, and it continues to be performed in many facilities. There are several reasons for this: First, the number of pancreatic transplants performed in Japan was extremely small, and comparative studies could not be conducted. The organ transplantation law was amended, and the number of transplants increased, making it possible to plan comparative trials. Second, blood-flow measurement using the pulse Doppler method, a well-known blood-flow measurement method after transplantation, makes it difficult to visualize the blood flow because the graft is located deep in the abdominal cavity and the blood flow is slow. Measurement by catheter insertion is highly invasive for routine measurements. Most pancreatic transplants involve kidney transplants, and the use of iodine-based contrast media imposes a burden on the kidneys, making pancreatic graft perfusion difficult to evaluate. To address this problem, a contrast-enhanced ultrasound (CEUS) was used to quantify the tissue perfusion in pancreatic grafts, and its usefulness was reported [11,12].

This study is a randomized prospective trial investigating the effect of CHA reconstruction on the frequency of complications, endocrine function, and tissue perfusion.

## 2. Materials and Methods

### 2.1. Study Design

This study was a prospective, randomized, controlled, and parallel-arm clinical trial that assessed the impact of CHA reconstruction. It was designed to show the noninferiority of CHA non-reconstruction in graft survival. The primary endpoint was the graft survival rate at 1 month after transplantation (Tx). In addition, the complication rate, degree of tissue perfusion, graft endocrine function after transplantation, graft survival, and patient survival up to 1 year after Tx were examined. There are no reports showing the effectiveness of CHA reconstruction, and its contribution is unknown. Therefore, we determined the sample size for this study based on our past results. We reported that the survival rate of pancreas transplantation performed at our hospital was 93.2% at 1 month after Tx [1]. Common hepatic artery reconstruction was performed in all cases, and this value was used as the standard for CHA reconstruction. Pilot data of a small number of non-reconstructed cases showed 100% graft survival rate at 1 month; based on this, it was expected that the graft survival rate of CHA non-reconstruction would be equal to or higher than that of CHA reconstruction cases. Thus, it was hypothesized that 93% survival rate at 1 month would be obtained in both groups. We split the study period into a registration period of 3 years and an observation period of 4 years, and the lower limit of noninferiority was set to 80%, since the number of cases that can be performed is limited. Furthermore, the sample size was calculated by setting the power (1-β) to 80% and α = 0.05. As a result, it was derived that the number of cases required was 12 in both groups. After accounting for the excluded cases, we set the number of the study population to 15 cases in both groups.

All study participants were informed regarding the clinical trial prior to surgery, and written consent was obtained from the patients. This study was conducted in accordance with the Declaration of Helsinki, and the protocol was approved by the Ethics Committee of the Fujita Health University (HM16-287). Furthermore, this study was registered at the University Hospital Medical Information Network Clinical Trials Registry under UMIN000027213. This report was prepared in accordance with the CONSORT statement.

### 2.2. Study Population

Patients included in the study were between the age groups of 32 and 65 years and underwent pancreas transplantation at the Fujita Health University Hospital between August 2017 and November 2020. Exclusion criteria included the following: patients allergic to eggs as the perflubutane microbubbles used for CEUS contained egg components, an absence of backflow from the GDA, and patients whose donor arteries were thin and CHA could not be reconstructed.

**Figure 1:**
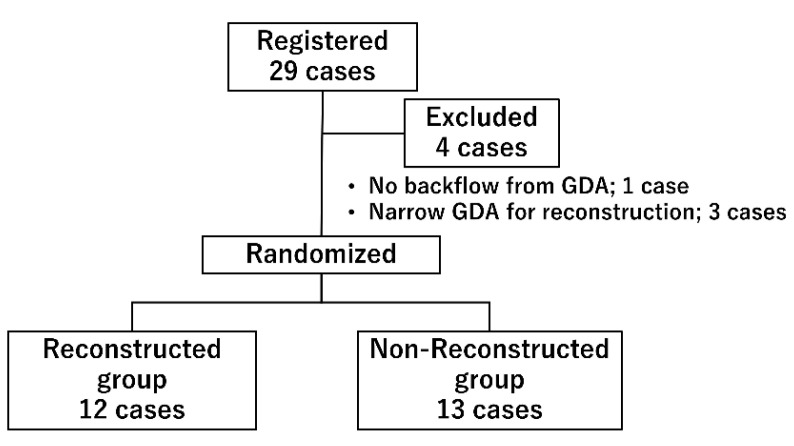
Study profile.

In this study, block randomization was performed every two cases by one investigator who was not a surgeon in charge of pancreas transplantation. The allocation was decided immediately before the transplantation, and the surgeon was informed. Out of the 29 registered cases, 25 cases were randomized into the non-reconstructed group and reconstructed group (Figure 1). Four cases were excluded: one due to the absence of backflow from the GDA, and three cases due to narrow GDAs, making it difficult to perform arterial reconstruction (all three patients received a pancreas from pediatric donors).

Twenty-nine patients were enrolled in this study. Four cases were excluded, and 25 cases were randomized and divided into 12 cases in the reconstruction group and 13 cases in the non-reconstruction group.

### 2.3. Transplantation Procedure

**Figure 2:**
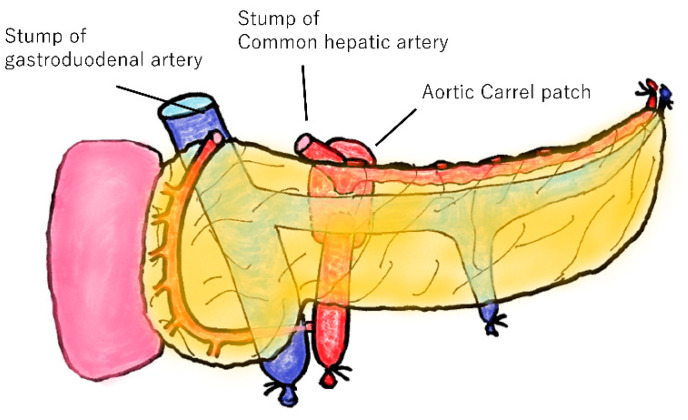
Typical pancreatic graft in Japan.

Transplant surgeries were performed at Fujita Health University Hospital. All pancreatic grafts were procured from brain-dead donors. A typical vascular dissection of a pancreatic graft is shown in Figure 2. The CHA was dissected in the middle, and the GDA was dissected near the bifurcation. In addition, the celiac trunk can also be used as a pancreatic graft, and an aortic Carrel patch can be formed together with the SMA. The portal vein was dissected at a height of 5 mm from the upper edge of the pancreas.

The gastroduodenal and common hepatic arteries are dissected. The celiac trunk is used for pancreatic grafts and can form an aortic Carrel patch along with the superior mesenteric artery.

In all cases, the CHA and SMA grafts were anastomosed to the external iliac artery as a Carrel patch, and the portal vein graft was anastomosed to the external iliac vein. The duodenum graft was anastomosed with the recipient’s ileum using Roux-en-Y anastomosis.

The decision to perform CHA reconstruction was made before surgery. It was performed in two ways: direct anastomosis was performed when the dissected donor CHA and GDA were in proximity, or an I-graft using a part of the donor iliac artery was performed when there was a distance between the two arteries. If not reconstructed, both the CHA and the GDA were ligated.

For prevention of graft thrombus, patients continuously injected heparin during 10 days from the operation. The dose of heparin is decided by activated clotting time (ACT). Target time of ACT is 180 s. After the end of continuous heparin administration, patients took edoxaban for 1 year.

### 2.4. Immunosuppression Protocol

Maintenance immunotherapy was initiated using tacrolimus, mycophenolate mofetil, and prednisolone. All patients also received induction therapy at the time of transplantation with basiliximab for simultaneous pancreas transplantation and thymoglobulin for pancreas transplantation after kidney transplantation/pancreas transplantation alone. The difference in the drugs administered is because of the Japanese healthcare system; basiliximab is only approved for induction therapy for kidney transplantation. Maintenance immunotherapy was adjusted according to the patient’s general condition and occurrence of side effects.

### 2.5. Evaluation Items

#### 2.5.1. Primary Endpoints and Secondary Endpoints

The primary endpoints were graft survival at 1-month after Tx; and secondary endpoints were the incidence of perioperative complications (especially duodenal perforation and graft venous thrombosis), graft survival at 1-year after Tx, patient survival at 1-month and 1-year after Tx, graft endocrine function at 1-month and 1-year post transplantation, and tissue perfusion immediately after transplantation. All examinations in the study were performed at Fujita Health University Hospital, and the data was collected by the investigators.

#### 2.5.2. Assessment of Graft Function

The patients underwent a glucagon stimulation test and a 75 g oral glucose tolerance test (OGTT) at 1-month and 1-year post transplantation. The glucagon stimulation test measured C-peptide levels (CPR) before and 6 min after loading. The amount of basal CPR and the amount of change in CPR (delta-CPR) were analyzed. In the OGTT, blood samples were collected before loading and 30, 60, 90, 120, and 180 min after loading, and blood glucose and insulin concentrations were measured. In addition to the value at each time point, the OGTT phenotype and area under the curve (AUC) were analyzed.

#### 2.5.3. Assessment of Tissue Perfusion

Pancreatic graft perfusion was measured using CEUS [12]. Perflubutane microbubbles were administered intravenously, and the contrast of the graft was recorded. In the recorded data, regions of interest were set for the portal vein and pancreatic parenchyma, and the difference in peak time was used as a baseline for tissue perfusion as Delta-Tp. CEUS was performed within 24 h after transplantation.

### 2.6. Statistical Analysis

The results are expressed as median and interquartile range. All statistical analyses were performed using EZR (Saitama Medical Center, Jichi Medical University, Saitama, Japan), which is a graphical user interface for R (The R Foundation for Statistical Computing, Vienna, Austria) [13]. Graft survival rate, which is the primary endpoint, and patient survival rate, were compared using the log-rank test. Other items were analyzed using the chi-square test or Mann–Whitney U test. Statistical significance was set at *p* < 0.05.

## 3. Results

### 3.1. Backgrounds of the Participents

Table 1 shows the backgrounds of both the groups. There was no difference in recipient backgrounds between the two groups. Donor age and body mass index tended to be lower in the non-reconstructed group than in the reconstructed group, but the difference was not significant.

### 3.2. Graft Survival and Complications

**Figure 3:**
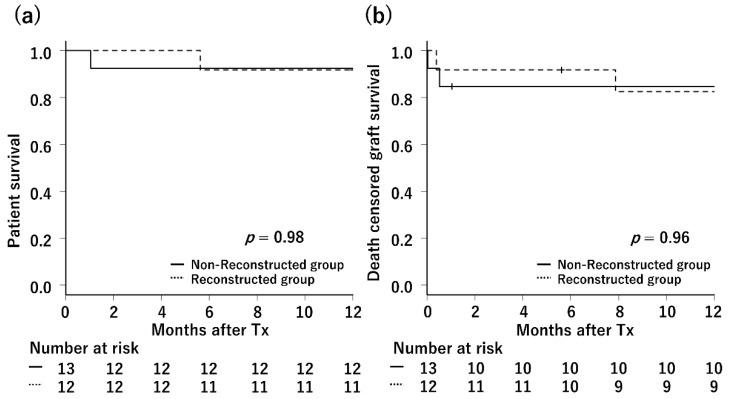
Kaplan–Meier curve for patient survival and graft survival. (**a**) One-year patient survival: The 1-year patient survival rates were 92.3% and 91.7% in the non-reconstructed and reconstructed groups, respectively. There was no significant difference in survival between the two groups. (**b**) One-year graft survival: The 1-year death censored graft survival rates were 83.3% and 82.5% in the non-reconstructed and reconstructed groups, respectively. There was no significant difference.

The 1-year patient survival rates of the non-reconstructed and reconstructed groups were 92.3% and 91.7%, respectively (Figure 3a). There was no statistical difference between the groups. There was one death in both groups, all of which were due to multiple organ failure caused by severe infections. The death-censored graft survival rates of the non-reconstructed and reconstructed groups were 83.3% and 82.5%, respectively (Figure 3b). There was also no statistical difference between the groups.

**Figure 4:**
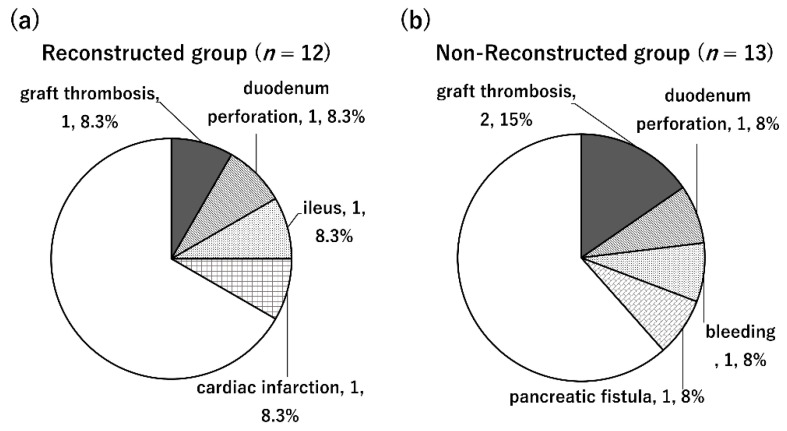
Complications after transplantation. (**a**) Complications in the non-reconstructed group: There were two cases of graft thrombosis and one case of duodenal perforation. In addition, bleeding and pancreatic juice leakage were observed in one case each. (**b**) Complications in the reconstructed group: There was one case of graft thrombosis and one case of duodenal perforation. In addition, one case of ileus and one case of myocardial infarction were observed.

Perioperative mortality was not observed in either group. Complications of grade 3 or higher according to the Clavien–Dindo classification occurred in 5/13 (38.5%) cases in the non-reconstructed group and 4/12 cases (33.3%) in the reconstructed group (Figure 4). The most common complication in this study was graft venous thrombosis, followed by duodenal perforation. Graft venous thrombosis occurred in 2/13 cases (15.4%) and 1/12 cases (8.3%) in the non-reconstructed and reconstructed groups, respectively. Graft duodenal perforation occurred in 1/13 cases (7.7%) and 1/12 cases (8.3%) in the non-reconstructed and reconstructed groups, respectively. There was no significant difference in the incidences of these complications between the two groups. In addition, bleeding, ileus, pancreatic fistula, and myocardial infarction were observed in one case each. Perioperative graft failure was observed in two cases in the non-reconstructed group and in one case in the reconstructed group, all of which were due to graft venous thrombosis.

### 3.3. Graft Function at 1 Month after Tx

**Figure 5:**
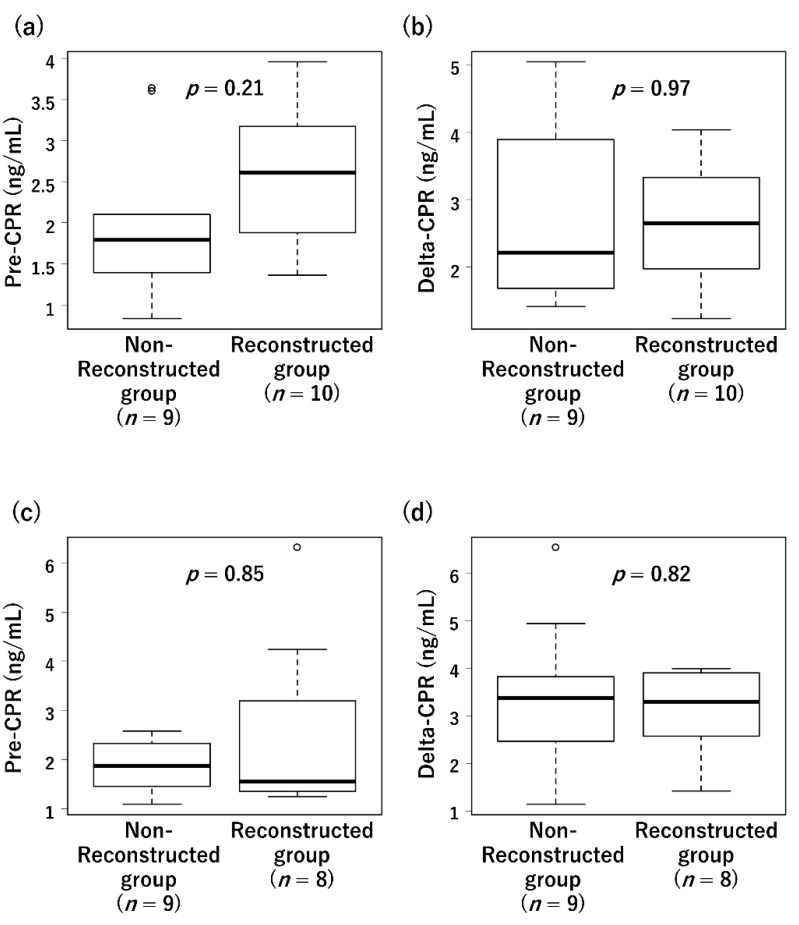
Results of glucagon stimulation test. (**a**) Pre-load C-peptide (CPR) at 1 month after transplantation: Pre-load CPR was lower in non-reconstructed group but not significantly different (the non-reconstructed group vs. the reconstructed group; 1.79 (1.39–2.10) vs. 2.61 (2.00–3.10), *p* = 0.211). (**b**) Delta-CPR at 1 month after transplantation: Delta-CPR of both the groups were comparable (the non-reconstructed group vs. the reconstructed group; 2.21 (1.68–3.89) vs. 2.65 (2.10–3.27), *p* = 0.968). (**c**) Pre-load CPR at 1 year after transplantation: Pre-load CPR was not significantly different between two groups (the non-reconstructed group vs. the reconstructed group were 1.72 (1.31–2.23) vs. 1.56 (1.37–2.67), *p* = 0.85). (**d**) Delta-CPR at 1 year after transplantation: There was no significant difference in delta-CPR between the two groups (the non-reconstructed group vs. the reconstructed group; 3.38 (2.47–3.82) vs. 3.30 (2.70–3.89), *p* = 0.82).

All 22 patients who achieved graft survival at 1 month after transplantation (Tx, in the Results section) achieved insulin withdrawal. The results of the glucagon stimulation test at 1 month after Tx are shown in Figure 5a,b. Pre-load CPR was not significantly different between the reconstructed and non-reconstructed groups (1.79 (1.39–2.10) vs. 2.61 (2.00–3.10), *p* = 0.21), and sufficient basal secretion was observed in both the groups. Delta-CPR was 2.21 (1.68–3.89) and 2.65 (2.10–3.27) in the non-reconstructed and reconstructed groups, respectively. There was no significant difference in delta-CPR (*p* = 0.97).

**Figure 6:**
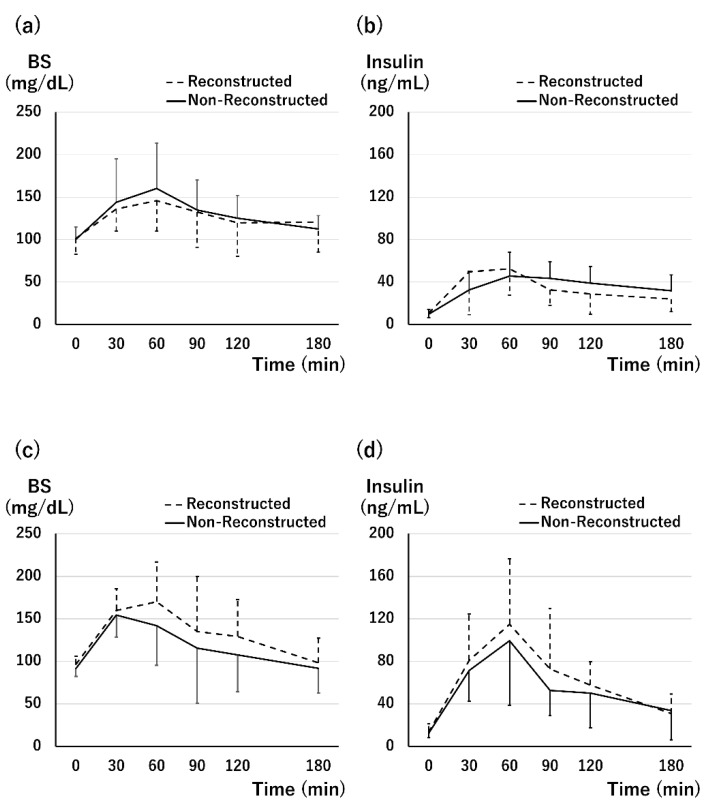
Results of oral glucose tolerance test. (**a**) Change in the blood glucose (BS) level at 1 month after transplantation: BS transition of both the groups was not significantly different during the test. The area under the curve (AUC) of BS level during the test was comparable between the two groups (the non-reconstructed group vs. the reconstructed group; 385.4 (321.6–424.6) vs. 365.4 (351.3–426.3), *p* = 0.82). (**b**) Change in the insulin level at 1 month after transplantation: The insulin peak was 30 min in the reconstructed group and 60 min in the non-reconstructed group. There was no significant difference in the value during the examination. The AUC of insulin level showed no difference between the two groups (the non-reconstructed group vs. the reconstructed group; 120.1 (80.9–139.8) vs. 106.8 (65.7–131.4), *p* = 0.67). (**c**) Change in the BS level at 1 year after transplantation: Both the groups showed sufficient BS transition. There was no significant difference in the values during the examination. The AUC of BS level during the test was comparable between the two groups (the non-reconstructed group vs. the reconstructed group; 331.3 (287.3–366.1) vs. 370.5 (343.0–488.4), *p* = 0.28). (**d**) Change in the insulin level at 1 year after transplantation: The changes in BS and insulin levels in both the groups were similar during test times. There was no difference in the peak insulin time between the two groups. The AUC of insulin level showed no difference between the two groups (the non-reconstructed group vs. the reconstructed group; 168.3 (112.8–194.8) vs. 186.7 (158.1–189.7), *p* = 0.69).

The OGTT at 1 month after Tx showed almost similar blood glucose (BS) transition (Figure 6a). The AUC of the BS level of the non-reconstructed group was comparable with that of the reconstructed group (the non-reconstructed group vs. the reconstructed group; 385.4 (321.6–424.6) vs. 365.4 (351.4–426.3), *p* = 0.82). The insulin peak of the non-reconstructed group was 60 min after loading, which was late compared with that of the reconstructed group (Figure 6b). No significant difference was observed in the AUC of insulin level (120.1 (80.9–139.8) vs. 106.8 (65.7–131.4), *p* = 0.67). In terms of insulin level, 5/8 cases (62.5%) in the non-reconstructed group and 6/9 cases (66.7%) in the reconstructed group showed a normal pattern. One patient in the non-reconstructed group (12.5%) and two in the reconstructed group (22.2%) showed a diabetic pattern.

### 3.4. Graft Function at 1 Year after Tx

Hemoglobin A1c levels at 1 year after Tx were 5.5% (5.1–5.6) in the non-reconstructed group and 5.3 (5.1–5.4)% in the reconstructed group, and there was no significant difference (*p* = 0.59).

Seventeen patients underwent a glucagon stimulation test 1 year after Tx. The pre-load CPR of the non-reconstructed and reconstructed groups were 1.72 (1.31–2.23) and 1.56 (1.37–2.67), respectively, and no significant difference was observed (*p* = 0.85, Figure 5c). There was no significant difference between the two groups in delta-CPR (the non-reconstructed group vs. the reconstructed group; 3.38 (2.47–3.82) vs. 3.30 (2.70–3.89), *p* = 0.82, Figure 5d). Compared with 1 month after Tx, responsiveness to the test did not change significantly and was sufficient in both groups.

The OGTT was performed in 18 patients. The changes in BS and insulin levels in both groups were comparable (Figure 6c,d). Normal results were observed in 8/10 cases in the non-reconstructed group and in 5/8 cases in the reconstructed group. However, 2/10 cases in the non-reconstructed group and 3/8 cases in the reconstructed group represented the impaired glucose tolerance, and there were no cases of diabetes mellitus. Furthermore, there was no difference in the frequency of phenotypes between the two groups (*p* = 0.61).

### 3.5. Tissue Perfusion

CEUS was performed in all 25 patients, and one patient was diagnosed with graft venous thrombosis and immediately underwent graft pancreatectomy. Delta-Tp(P-V) was measured in 24 patients. The difference between cases was large; the Delta-Tp(P-V) ranged from 1.83 s to 11.01 s. The Delta-Tp(P-V) of the non-reconstructed group was 4.68 s (3.49–7.09) and 4.16 s (3.33–7.05) in the reconstructed group, with no significant difference between the two groups (*p* = 1.0).

## 4. Discussion

CHA reconstruction has been performed in Japan for a long time. This study was the first to scientifically analyze the effects of CHA reconstruction and to evaluate tissue perfusion after pancreas transplantation. Our study confirmed that CHA reconstruction does not improve graft survival or reduce complications. This finding will have a significant impact on the surgical technique used for pancreas transplantation. There are many variations in the vascularization of the pancreas [10], and a part of the blood vessels is often excised during organ collection; therefore, revascularization of the graft is an important procedure [14]. Apart from CHA reconstruction, various procedures maintain good blood flow in pancreatic grafts [15,16]. To perform these procedures, outstanding skills for anastomosing small blood vessels are required. Furthermore, increasing the number of vascular anastomosis sites increases the risk of complications, such as bleeding. It also prolongs back table surgery time. Despite these disadvantages, CHA reconstruction is recommended to increase blood flow to the pancreatic head. However, the results of this study suggest that blood flow from the SMA is sufficient to maintain the graft pancreatic head.

As Pinchuk et al. stated, the arteries in the pancreas form a complex arcade, and in some cases, imaging studies of the entire pancreas can be obtained by injecting a contrast medium through the splenic artery [17]. However, some grafts do not have a sufficiently developed arcade. In this study, one out of 30 patients did not have sufficient backflow from the GDA stump. This case was excluded during registration, and a CHA reconstruction was performed. It is necessary to keep in mind that underdeveloped arcades exist in some graft pancreas. If CHA reconstruction is not performed in such cases, localized blood flow in the pancreatic head region may decrease, which may lead to serious complications, such as duodenal perforation. Therefore, checking the backflow from the GDA stump on the back table is essential. If sufficient spillage is not observed, CHA reconstruction should be performed without hesitation. It is also important to be careful not to cut off the branches of the GDA or IPDA when collecting or trimming organs. This study included pediatric donors, of whom three had extremely narrow GDAs, and therefore, reconstruction was difficult. Fortunately, all of them showed sufficient GDA backflow and did not require the use of difficult reconstruction techniques, and the pediatric graft function was sufficient. This is a useful option in Japan where the number of donors is limited [18], while carefully considering the potential for revascularization. In addition to reconstruction of the proper hepatic artery, as performed in this study, a useful revascularization method using the right gastroepiploic artery has also been reported [16].

Duodenal perforation, a major complication after pancreas transplantation, may be associated with ischemia [19,20,21]. Orsenigo et al. clarified that the transplanted duodenum had a significantly lower blood flow than the recipient small intestine using intraoperative laser Doppler flowmetry [20]. Whether this reduced blood flow is a risk factor for duodenal complications remains unclear, as it is not known if this blood flow is sufficient or how it changes over time. However, the results of this study revealed that CHA reconstruction did not improve duodenal blood flow. The perfusion in the duodenum itself may be decreased, but it is difficult to reduce the risk of duodenal complications by improving the blood flow, and a different perspective is needed. The incidence of duodenal perforation in this study was approximately 8% (two cases) with or without CHA reconstruction, which is the same as previously reported [2,4]. The donors in these two cases were relatively young, <45 years of age. In contrast, it should be noted that both patients who experienced perforation had a long history of dialysis. Interestingly, three of the participants in this study had undergone dialysis for more than 10 years, two of whom experienced a duodenal perforation. Hence, it can be inferred that long-term dialysis history is a risk factor for duodenal perforation.

This study had some limitations. First, the number of cases was small. The number of pancreatic transplants at our hospital is approximately 10 cases per year, and although this study had a sufficient time period of 3 years, approximately 30 cases were registered. Another limitation is that this study was an open-label study because it was related to surgical techniques. No significant difference was observed between the two groups, suggesting that the study was of sufficient scale to assess the effects of CHA reconstruction.

In conclusion, the results of this study show that in cases where pancreatic arcades were sufficiently developed, no increase in complications and no decrease in survival rate were observed due to non-reconstruction of the CHA. In addition, no decrease in endocrine function was observed. These findings show that in pancreas transplantation, CHA reconstruction is indicated for limited cases and is not necessary as a standard procedure.

## Figures and Tables

**Table 1:** Recipient and donor background of both the groups.

		Non-Reconstructed Group (*n* = 13)	Reconstructed Group (*n* = 12)	*p*-Value
Recipient factor				
	Age (years)	48.2 ± 9.5	47.9 ± 8.3	0.93
	Sex (male, %)	5 (38.5)	5 (41.7)	1
	Duration of DM (yr)	30.6 ± 10.0	26.8 ± 7.7	0.29
	Duration of HD (yr)	6.3 ± 5.8	4.9 ± 3.6	0.52
Donor factor				
	Age (years)	35.5 ± 17.8	42.2 ± 12.6	0.30
	Sex (male, %)	5 (38.5)	8 (66.7)	0.24
	BMI (kg/m^2^)	20.7 ± 4.3	23.7 ± 3.5	0.067
	Cause of death (%)			0.69
	non-CVD	7 (53.8)	8 (66.7)	
	CVD	6 (46.2)	4 (33.3)	
	Episode of CPA (%)	10 (76.9)	6 (50.0)	0.23
	Serum creatinine (mg/dL)	0.80 ± 0.70	0.79 ± 0.31	0.97
	HbA1c (%)	5.58 ± 0.19	5.67 ± 0.29	0.36
Operative procedure (%)				1
	PAK	1 (7.7)	1 (9.1)	
	SPK	12 (92.3)	11 (90.9)	
Cold ischemia time (hr)		13.2 ± 4.1	12.7 ± 1.9	0.69

DM, diabetes mellitus; HD, hemodialysis; BMI, body mass index; CVD, cerebrovascular disorder; CPA, cardiopulmonary arrest; PAK, pancreas transplantation after kidney transplantation; SPK, simultaneous pancreas kidney transplantation.

## Data Availability

The data presented in this study are available on request from the corresponding author. The data are not publicly available due to restrictions as a result of their containing information that could compromise the privacy of research participants.
